# Integrative analysis revealed the role of glucagon-like peptide-2 in improving experimental colitis in mice by inhibiting inflammatory pathways, regulating glucose metabolism, and modulating gut microbiota

**DOI:** 10.3389/fmicb.2023.1174308

**Published:** 2023-05-16

**Authors:** Dongyue Li, Yanhong Gao, Lanrong Cui, Yang Li, Hao Ling, Xin Tan, Hongyu Xu

**Affiliations:** ^1^Department of Gastroenterology, First Affiliated Hospital of Harbin Medical University, Harbin, Heilongjiang, China; ^2^Department of Gastroenterology, General Hospital of Northern Theater Command, Shenyang, Liaoning, China

**Keywords:** glucagon-like peptide-2, ulcerative colitis, intestinal flora, inflammatory pathways, glucose metabolism

## Abstract

**Introduction:**

Ulcerative colitis (UC) is an inflammatory bowel disease characterized by recurrent and remitting inflammation of the mucosa of the colon and rectum, the incidence of which is on the rise. Glucagon-like peptide-2 (GLP-2) is a newly discovered neurotrophic factor, but its efficacy and mechanism of action in UC remain unclear. In this study, we investigated the protective effects and potential targets of GLP-2 on dextran sodium sulfate (DSS)-induced UC in mice through integrative analysis.

**Methods:**

The effects of GLP-2 on UC were assessed by calculating the disease activity index, colonic mucosal damage index, and pathological histological scores. Enzyme-linked immunosorbent assay (ELISA) and immunohistochemistry were used to detect the expression of GLP-2, nuclear factor kappa-B (NF-κB), interleukin-6 (IL-6), and signal transducer and activator of transcription-3 (STAT3). The 16SrRNA gene was used to detect changes in gut microbiota in mouse colonic tissues, and oral glucose tolerance test (OGTT) blood glucose levels were used to analyze the differences in flora.

**Results:**

The results showed that GLP-2 could reduce the inflammation of UC mice, which may be achieved by inhibiting the potential targets of NF-κB, and Janus kinase (JAK)/STAT3 inflammatory pathways, regulating sugar metabolism, increasing dominant species, and improving microbial diversity.

**Discussion:**

This study provides new insight into the potential of GLP-2 for achieving more ideal UC treatment goals in future.

## 1. Introduction

Ulcerative colitis (UC) is a chronic inflammatory disease of the rectum and colon characterized by mucosal inflammation (Abdulrazeg et al., 2019). The exact pathogenesis of UC is not fully understood and is generally thought to be related to the interaction between the environment, the immune system, the intestinal flora, and genetic susceptibility (Du and Ha, 2020). The intestinal flora plays an important role in maintaining the integrity of the intestinal mucosa, and disruption of the intestinal mucosal barrier and bacterial invasion leads to intestinal inflammation (Guo et al., 2020). UC is characterized by a dysregulated inflammatory response, manifested by invasion of inflammatory cells into the lamina propria and excessive release of pro-inflammatory cytokines. There is growing evidence that pro-inflammatory cytokines and the JAK/STAT3 pathway play a critical role in the pathogenesis of human UC and experimental colitis (Cordes et al., 2021). NF-κB, a major regulator of innate immunity and inflammatory responses, also upregulates pro-inflammatory cytokine expression in UC, triggering an inflammatory cascade response (Lin et al., 2019). STAT3 also activates NF-κB and induces the release of the pro-inflammatory mediator IL-6, which reactivates the JAK/STAT3 pathway, resulting in a malignant pro-inflammatory cycle (Fan et al., 2013). These events lead to the persistence of inflammation in the colonic mucosa, hindering mucosal regeneration, leading to tissue damage and ultimately to the progression of the disease toward inflammatory-cancerous transformation (Yao et al., 2019).

The currently used pharmacological interventions aim to counteract the characteristic episodes of intestinal inflammation. The most effective drugs are corticosteroids and tumor necrosis factor-α (TNF-α) inhibitors. However, the former cannot be used for a long time due to severe side effects, and the latter has a considerable number of primary and secondary non-responders (Ungaro et al., 2017). Therefore, it is crucial to find new and safer treatments.

Patients with ulcerative colitis also commonly present with abnormalities in glucose metabolism, which can be very harmful in patients with ulcerative colitis, limiting the type of elemental diet and enteral nutrition on the one hand, and creating obstacles to treatment (e.g., hormone application or emergency surgery) on the other hand. Modern studies proved that abnormalities of glucose metabolism, such as ulcerative colitis, are diseases closely related to intestinal flora, so we speculated whether changes in certain flora affect both inflammatory and glucose metabolism factors. In the process of searching for the association between the two, we found a key factor, glucagon-like peptide-2 (GLP-2), a peptide hormone with multiple beneficial effects on the intestine and a key target for glucose regulation, which has been successfully applied to the treatment of diabetes with established efficacy in clinical medicine. Therefore, this experiment introduced GLP-2 as one of the interfering factors and observation indexes, hoping to clarify the relationship between intestinal flora and the distribution and severity of ulcerative colitis, as well as to have unexpected gains in the relationship between ulcerative colitis and abnormal glucose metabolism and GLP-2 and the internal environment of flora.

The rapid development and widespread application of high-throughput sequencing technology in recent years have elevated our study of bacteria in the gut to the level of big data on the Internet, making research more efficient. Machine learning methods, especially the deep learning methods, have been applied to various fields of bioinformatics, such as human ether-a-go-go-related gene (hERG) blocker prediction (Wang T. et al., 2023), metabolite–disease association prediction (Sun et al., 2022), miRNA–lncRNA interaction prediction (Wang et al., 2022), and single-cell multimodal data (Hu et al., 2023). These studies have contributed in part to the important role of high-throughput measurements in solving elusive biological and medical problems.

GLP-2 is an enterotrophic hormone released from enteroendocrine L cells that exerts an indirect gut-protective effect through different mediators. However, *in vivo* GLP-2 is rapidly degraded by dipeptidyl peptidase (DPP)-IV, reducing its potential physiological effects; therefore, inhibition of DPP-IV to extend the half-life of GLP-2 may have an ameliorative effect on UC. Sitagliptin is the first DPP-IV inhibitor approved by Food and Drug Administration (FDA) for the treatment of type 2 diabetes, and a large amount of preclinical and clinical data, as well as post-marketing monitoring data, demonstrate that it is an effective and safe DPP-IV inhibitor. However, while sitagliptin prolongs the half-life of GLP-2 *in vivo*, it also inhibits the degradation of GLP-1, and it is unknown whether GLP-1 exerts an enteroprotective effect in addition to its known role in regulating blood glucose and its newly discovered neuroprotective effect. In this experiment, DSS was selected to construct a mouse model of UC. Because clinical ulcerative colitis patients are often complicated with pathogenic bacteria infection, enterotoxigenic *Escherichia coli* (ETEC) was introduced to aggravate intestinal inflammation in mice, better simulate the condition of clinical ulcerative colitis patients, and further explore the efficacy and possible mechanism of GLP-2 in the treatment of UC.

## 2. Materials and methods

### 2.1. Reagents

DSS with a molecular weight of 36–50 kDa was purchased from MP Biomedicals. Sitagliptin was obtained from Sigma. ETEC was purchased from Beina Chuanglian Biotechnology Institute (Beijing, China). Mouse GLP-2, NF-κB p65, IL-6, STAT3 ELISA kits were purchased from Jiangsu Meimian industrial Co., Ltd. Rabbit anti-GLP-2, anti-NF-κB p65, anti-STAT3 polyclonal antibodies were purchased from Jiangsu Meimian industrial Co., Ltd. Fecal occult blood reagent was purchased from Shanghai Wei dysprosium Biotechnology Co., Ltd. Mouse blood glucose tester was purchased from Shenyang Jingdong Hongjian Trading Co., Ltd.

### 2.2. Animals

A total of 40 specific pathogen-free (SPF) female C57BL/6 mice aged 6–8 weeks and weighing 18–20 g were purchased from Liaoning Changsheng Biotechnology Co., Ltd., animal quality license number: SCXK (Liao) 2020-0001. All mice were maintained under specific pathogen-free conditions in Animal Laboratory Center of the First Affiliated Hospital of Harbin Medical University. The animal experiments complied with the ARRIVE guidelines and were approved by the Animal Ethics Committee of the First Affiliated Hospital of Harbin Medical University.

C57BL/6J mice were acclimatized and fed for 1 week, and the ear tag clips were well marked and randomly divided into four groups: blank control group (*n* = 10), DSS group (*n* = 10), DSS+ETEC group (*n* = 10), and DSS+ETEC+Sitagliptin (30 mg/kg) group (*n* = 10). Except for the blank control group, all groups of mice were given 2% DSS ad libitum from day 0 to 7. The mice in each group were given 0.2 ml ETEC (concentration 2.27 × 10^8^ CFU/ml) or sterile saline by gavage on the 3rd day of modeling. 0.1 ml 0.6% sitagliptin or sterile saline by gavage was given to each group from 0 to 13 days. The body weight, fecal occult blood, and water consumption of mice were recorded daily during the experiment. The OGTT test was performed on mice on the 3rd and 7th days of modeling. On the 13th day, 4% chloral hydrate was given intraperitoneally to each group of mice according to their body weight (0.1 ml/10 g), and then, blood was removed from the eyes and dissected to obtain colonic tissue.

### 2.3. DAI score

Mice were scored according to daily body weight change, fecal characteristics, and bleeding, and the disease activity index (DAI) score was obtained by dividing the three total scores by three. The specific scoring criteria are shown in Table 1.

**Table 1:** Disease activity index (DAI) scores.

**Weight change**	**Score**	**Stool consistency**	**Score**	**Bleeding stool**	**Score**
No change in body weight	0	Normal	0	Normal	0
Decrease of 1-5%	1				
Down 5-10%	2	loose and soft	2	Positive occult blood	2
Down 10-15%	3				
Decrease >15%	4	Watery	4	Dominant bleeding	4

### 2.4. CMDI and pathological histological score

The colon was cut along the longitudinal axis of the mesentery to observe the degree of colonic edema, ulceration, and other damage, according to the literature (Xie et al., 2022). The colonic mucosal damage index (CMDI) score was performed. The contents were removed by flushing with PBS and blotted with filter paper, and about 1 cm of anal side tissue was cut, fixed with sufficient amount of 10% formalin for 24 h or more, routinely embedded in paraffin, sectioned, stained with hematoxylin–eosin (HE), and observed under the microscope, and the pathological histological score was performed according to Table 2.

**Table 2:** Pathological histological scoring criteria.

**Part**	**Performance and Rating**
Mucosal epithelium	Ulcer formation: none (0); mild surface (1); moderate (2); extensive total (3)
Crypt	Mitotic activity: lower 1/3 (0); mild to moderate 1/3 (1); moderate to moderate 1/3 (2); upper 1/3 (3) Neutrophil infiltration
Mucosal lamina propria	Mucus Defects Plasma cell infiltration Neutrophil infiltration Vascular formation Cellulose deposition: none (0); restricted to mucosal layer (1); submucosal layer (2); wall permeability (3)
Submucosa	Neutrophil infiltration Edema

### 2.5. Oral glucose tolerance test

OGTT test method: (1) The mice fasted the night before the OGTT test but were given appropriate drinking water to ensure that the mice fasted for 12 h; (2) on the day of the test, the fasting blood glucose of each mouse was measured according to the above blood glucose measurement method; (3) after completing the fasting blood glucose measurement, each mouse was gavaged with 0.08 ml of 50% glucose solution; (4) after waiting for 2 h, the blood glucose values of the mice were measured again to complete the OGTT test.

### 2.6. Enzyme-linked immunosorbent assay

The blood was taken from the eyes of mice at 0.6–0.8 ml, centrifuged at 4000 r/min for 20 min, and the serum of mice was separated. The contents of GLP-2, NF-κB p65, IL-6, and STAT3 in the serum of each group of mice were determined. The dilution of standards, addition of samples, warming, liquid preparation, washing, addition of enzyme, and then addition of color developer were performed sequentially according to the instructions of the kit, and finally, the reaction was terminated to determine the optical density (OD) value and to calculate the actual concentration of the samples. Each sample to be measured was repeated twice, and the final average was taken.

### 2.7. Immunohistochemistry

After mouse colon tissue was paraffin-embedded and sectioned, the expression of GLP-2, NF-κB p65, and STAT3 was observed after sequential dewaxing, hydration, antigen repair, blocking, primary antibody, secondary antibody incubation, diaminobenzidine (DAB) color development, hematoxylin re-staining, dehydration, and sealing. A total of five high magnification fields were randomly selected for each section, 100 cells were counted in each high magnification field, the percentage of positive cells were counted, and the data were expressed as $\bar{\text{x}}$ ±S. The positive cells were divided into 3 grades according to their number and degree of color development: negative expression (−), that is, the number of positive cells < 10%, and the color intensity was light yellow; Expression is weakly positive (+), that is, the number of positive cells is 10%–25%, and the color intensity is yellow to brown; The expression is strongly positive (+++), that is, the number of positive cells is >50%, and the color intensity is brownish yellow to tan; The positive expression (++) is in between.

### 2.8. 16SrRNA sequencing

A total of five mice each from the blank control group, DSS group, and GLP-2 group were selected for DNA purity and concentration detection by NanoDrop2000 assay, and DNA integrity detection by agarose gel electrophoresis, primers were synthesized, PCR amplification was performed based on the variable region of 16SrRNA gene V3-V4, and the purified products were IlluminaPE300 sequencing. After quality control filtering of the raw data, operational taxonomic unit (OTU) clustering analysis was performed according to 97% similarity. Based on the results of OTU clustering analysis, diversity index analysis, detection of sequencing depth, and statistical analysis of community structure at each taxonomic level based on taxonomic information were performed.

### 2.9. Statistical analysis

Data were analyzed using SPSS 26. 0 and GraphPad Prism 8 statistical package. Differences in data between multiple groups for quantitative data were analyzed by one-way ANOVA, and the results were expressed as $\bar{\text{x}}$ ± S. The rank data were analyzed by chi-square test and plotted using GraphPad Prism 8. The intestinal flora data were statistically analyzed using the BioCloud platform of Shanghai Meiji Biomedical Technology Co. *P* < 0.05 means that the differences were statistically significant.

## 3. Results

### 3.1. Effect of GLP-2 intervention on the severity of mice

#### 3.1.1. DAI scores of mice in each group

**Figure 1:**
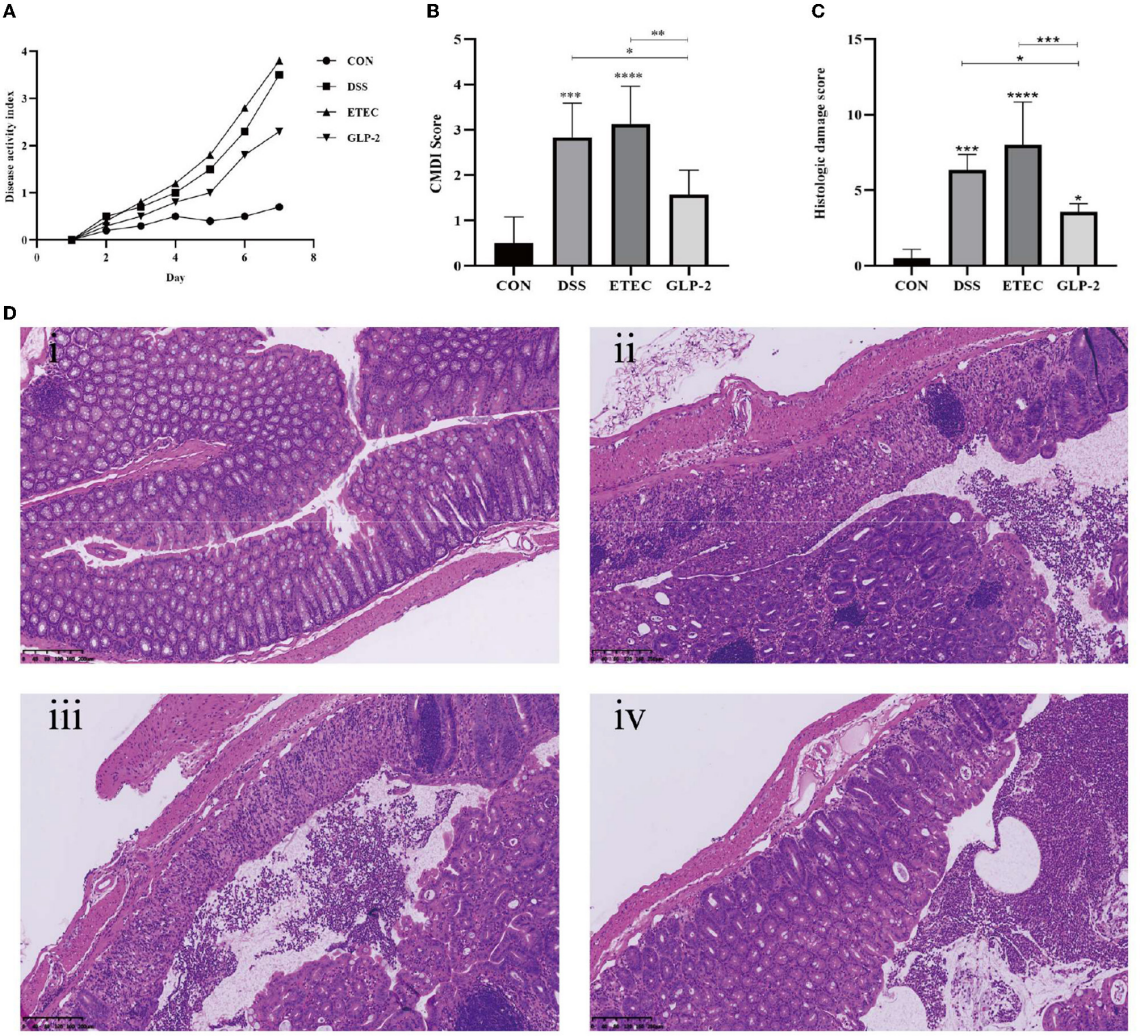
Effect of GLP-2 intervention on the severity of mice. **(A)** DAI scores of mice in each group. **(B)** Colonic mucosal damage index (CMDI) scores of mice in each group. **(C)** Pathological histological scores of mice in each group. **(D)** HE staining of colonic tissues of mice in each group i, ii, iii, and iv are in the order of CON, DSS, ETEC, and GLP-2 groups. ^*^P < 0.05; ^**^P < 0.01; ^***^P < 0.001; ^****^P < 0.0001.

Compared with the blank group, the DAI scores of DSS, ETEC, and GLP-2 groups were elevated; compared with the DSS group, the DAI scores of mice in the ETEC group were elevated, and the DAI scores in the GLP-2 group were decreased; compared with the ETEC group, the DAI scores in the GLP-2 group were decreased, but the differences were not statistically significant (see Figure 1A).

#### 3.1.2. CMDI scores for each group of mice

Compared with the blank group, mice in the DSS group had congested and edematous colonic tissue, rough mucosa, erosion, or ulceration, the damage index was significantly higher, and the difference was statistically significant (*P* < 0.001); mice in the ETEC group had necrosis and inflammation on the mucosal surface of colonic tissue and thickened intestinal wall, the damage index was significantly higher, and the difference was statistically significant (*P* < 0.0001). Compared with the DSS group, mice in the ETEC group had heavier colonic tissue damage and higher CMDI score, but the difference was not statistically significant (*P* > 0.05); mice in the GLP-2 group had mild colonic tissue congestion and edema, the damage index was significantly lower, and the difference was statistically significant (*P*<*0*.05); compared with mice in the ETEC group, mice in the GLP-2 group had significantly lower damage index, and the difference was statistically significant (*P* < 0.01) (see Figure 1B).

#### 3.1.3. HE staining and pathological histological scoring of colonic tissue in each group of mice

Compared with the blank group, the colonic histopathology scores of mice in the DSS group were significantly higher (*P*<*0*.001) and also significantly higher in the ETEC group (*P* < 0.0001); compared with the DSS group, the colonic histopathology scores of mice in the ETEC group were higher, but the difference was not statistically significant (*P* > 0.05), and the colonic histopathology scores of mice in the GLP-2 group were significantly lower. The colonic histopathology scores of mice in the GLP-2 group were significantly lower compared with the ETEC group (*P* < 0.05); the colonic histopathology scores of mice in the GLP-2 group were significantly lower compared with the ETEC group (*P* < 0.001) (see Figure 1C).

HE staining showed that the colon wall of control mice was intact, with normal morphology and number of cup cells and crypt cells, and few inflammatory cells. The DSS and ETEC groups caused a series of histopathological changes, including mucosal damage and necrosis, submucosal inflammatory cell infiltration, edema, and vascular congestion. The GLP-2 group of mice showed reduced histological changes and inflammatory cell infiltration in the colon and reduced local edema and vascular congestion (see Figure 1D).

### 3.2. Serum ELISA assay for each group of mice

**Figure 2:**
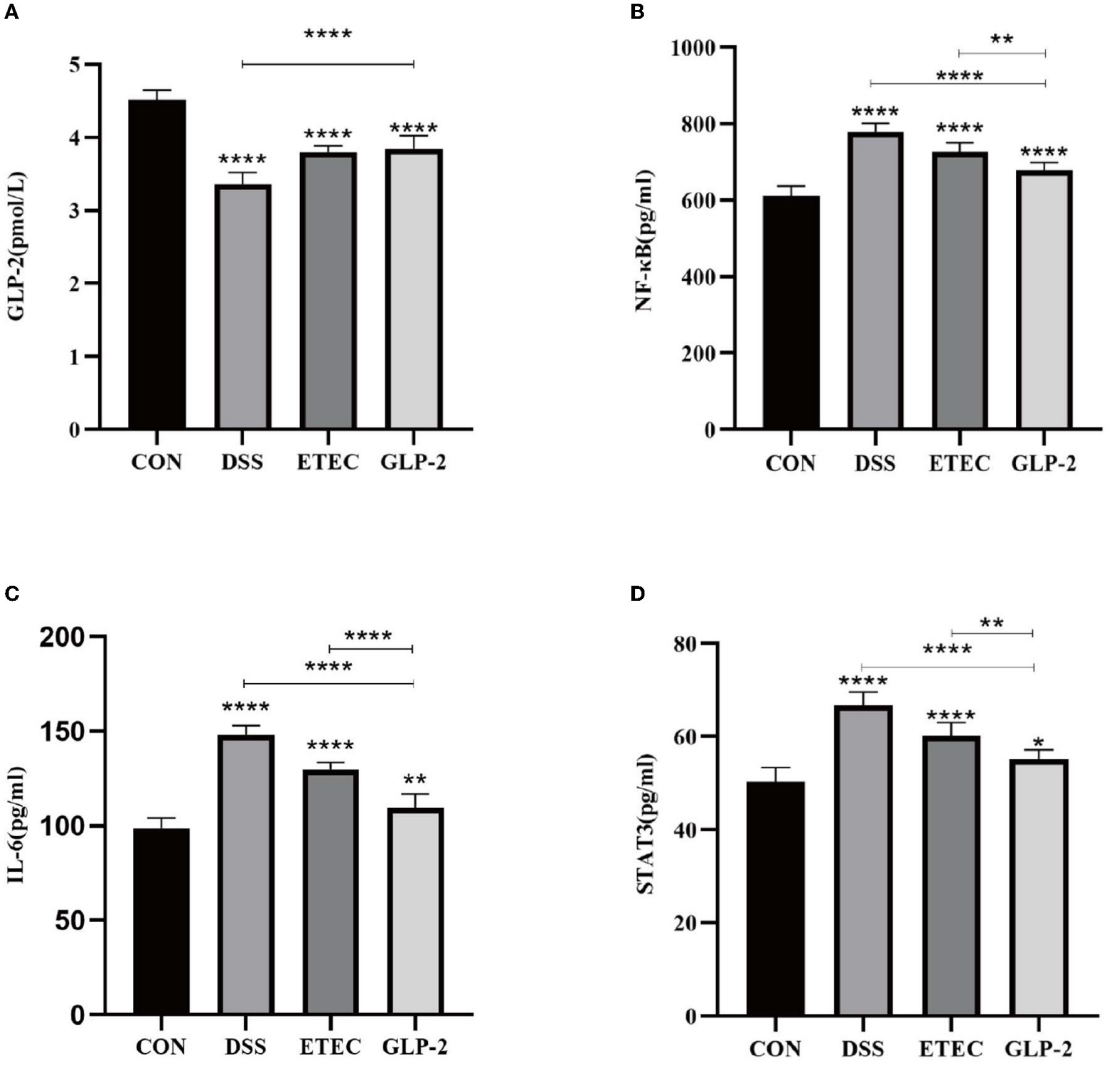
Serum ELISA assay for each group of mice. **(A)** Serum GLP-2 levels in each group of mice. **(B)** Serum NF-κB levels in each group of mice. **(C)** Serum IL-6 levels in each group of mice. **(D)** Serum STAT3 levels in each group of mice. *P < 0.05; **P < 0.01; ****P < 0.0001.

We listed the results of ELISA assay of GLP-2, NF-κB, IL-6, and STAT3 in the serum of mice in each group (see Table 3). Compared with the blank group, the serum GLP-2 values of mice in the DSS and ETEC groups were significantly lower (*P* < 0.0001), and NF-κB, IL-6, and STAT3 values were significantly higher (*P* < 0.0001); compared with the DSS group, the GLP-2 group mice had significantly higher GLP-2 values (*P* < 0.0001), and NF-κB, IL-6, and STAT3 values were significantly decreased (*P* < 0.0001); compared with ETEC group, NF-κB, IL-6, and STAT3 values in serum of mice in GLP-2 group were significantly decreased (see Figure 2).

**Table 3:** ELISA for GLP-2, NF-κB, IL-6, and STAT3 in the serum of each group of mice (x ± S).

**Group**	**Quantity**	**GLP-2 (pmol/L)**	**NF-κB (pg/ml)**	**IL-6 (pg/ml)**	**STAT3 (pg/ml)**
Control group	8	4.51 ± 0.14	611.30 ± 25.52	98.74 ± 5.27	50.39 ± 2.97
DSS group	8	3.36 ± 0.16^*^	779.80 ± 21.53^*^	148.0 ± 4.85^*^	66.71 ± 2.84^*^
ETEC Group	8	3.80 ± 0.09^*^	726.80 ± 23.31^*^	129.7 ± 3.78^*^	60.21 ± 2.84^*^
GLP-2 group	7	3.85 ± 0.18^*#^	677.60 ± 21.50^*#&^	109.7 ± 7.15^#∧^	55.10 ± 2.08^*#&*^

^*^P < 0.0001 vs. blank control group; ^#^*P* < 0.0001 vs. DSS group; ^&^*P* < 0.01 vs. ETEC group; ^∧^*P* < 0.0001 vs. ETEC group.

### 3.3. Immunohistochemical detection of colonic tissue in each group of mice

**Figure 3:**
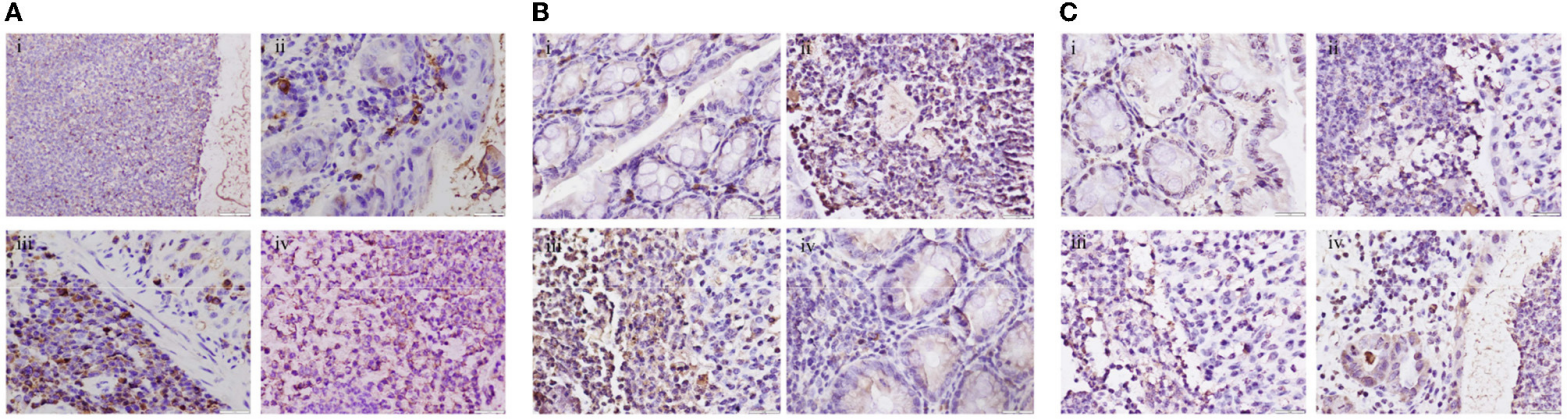
Immunohistochemical detection of colonic tissues of mice in each group (400x). **(A)** Expression levels of GLP-2 in colonic tissues of mice in each group. **(B)** Expression levels of NF-κB in colonic tissues of mice in each group. **(C)** Expression levels of STAT3 in colonic tissues of mice in each group i, ii, iii, and iv are in the order of CON, DSS, ETEC, and GLP-2 groups.

Compared with the blank group, the GLP-2 positive expression rate was significantly lower (*P*<*0*.05) and the NF-κB and STAT3 positive expression rates were significantly higher (*P* < 0.05) in the colon tissue of mice in the DSS and ETEC groups; compared with the DSS group, the GLP-2 positive expression rate was significantly higher (*P* < 0.05) and the NF-κB positive expression rate in the GLP-2 group was lower. The GLP-2 positive expression rate was significantly higher (*P*<*0*.05), NF-κB positive expression rate was lower (*P* > 0.05), and STAT3 positive expression rate was significantly lower (*P*<*0*.05) in the GLP-2 group compared with the ETEC group (see Figure 3).

### 3.4. Relationship between intestinal flora and inflammation and abnormal glucose metabolism

#### 3.4.1. Blood glucose results and grouping of samples for colony sequencing

We performed OGTT experiments on 10 mice each from the blank control group, DSS group, and GLP-2 group and concluded that the blood glucose in the DSS group was higher than that in the control group at OGTT2h (*P* = *0*.002), which was statistically significant; the blood glucose in the GLP-2 group was lower than that in the DSS group at OGTT2h, and the glucose tolerance was improved (*P* = 0.0018), which was statistically significant (see Table 4).

**Table 4:** Sequencing of blood glucose results in three groups of mice.

**Group**	**OGTT/(mmol/L)**
Control group (*n =* 10)	7.16 ± 0.89
DSS group (*n =* 10)	9.16 ± 1.57^*^
GLP-2 group (*n =* 10)	7.05 ± 1.62^#^

^*^P < 0.01 vs. blank control group; ^#^*P* < 0.01 vs. DSS group.

For the OGTT2h results, five mice in each group were screened, three with normal blood glucose and two with abnormal blood glucose for colony 16SrRNA sequencing analysis (only one with normal blood glucose and four with abnormal blood glucose in the DSS group), and grouped according to the requirements of raw letter analysis, with the normal group labeled as CON, the GLP-2 group as test1, the DSS group as test2, and the normal blood glucose (n) and abnormal blood glucose (C) groups.

#### 3.4.2. Species annotation of samples and assessment results

##### 3.4.2.1. OTU analysis

**Figure 4:**
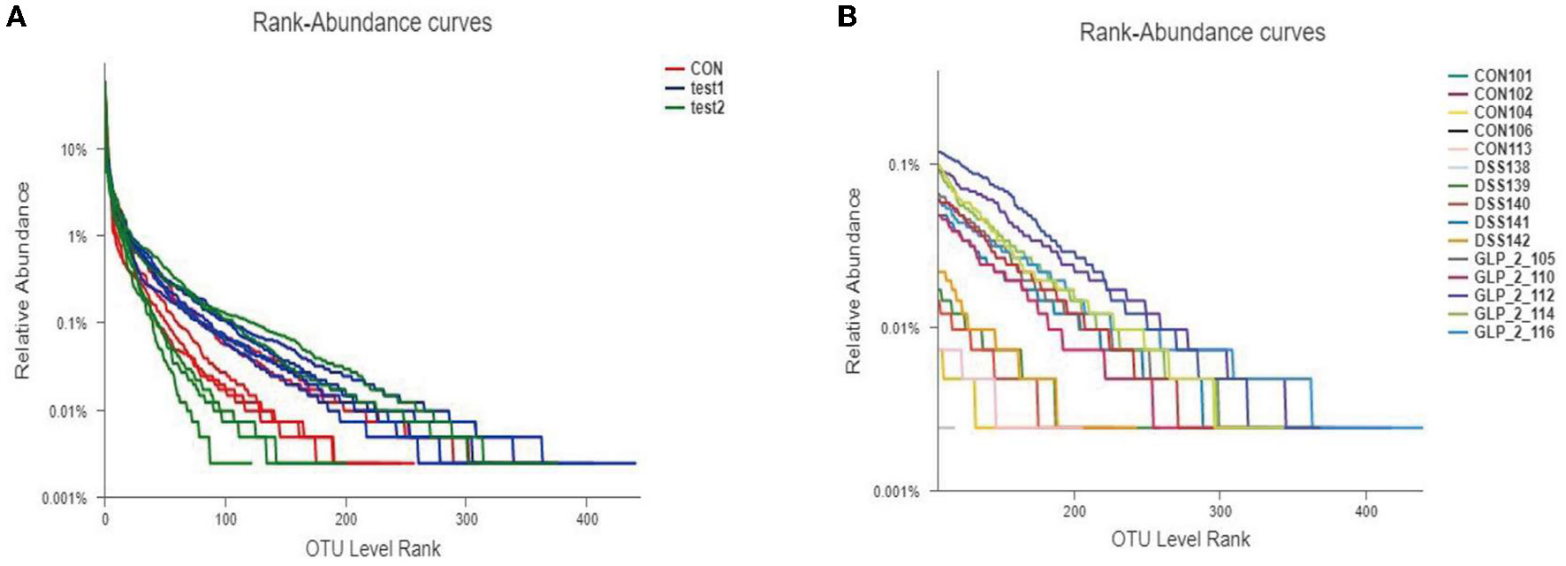
Rank-abundance curve of the sample. Different colors represent different groupings, the horizontal coordinates represent the number of flora in OTU clusters, the vertical coordinates represent the relative percentages of flora, and the length of the extension of the curve represents the total number of bacteria. **(A)** The rank-abundance curve in the group samples. **(B)** The rank-abundance curve in the detailed samples.

We clustered the colony information of the 15 samples sequenced and drew rank-abundance curves as shown in Figure 4; the results can be seen in the grouping curve (as shown in Figure 4A); test1 (GLP-2 group) curve declined the smoothest and extended the longest in the transverse coordinate, indicating that the number of species and diversity of the colony are higher; test2 (DSS group) curve declines steep value, the transverse coordinate is short in shape, which indicates that the number of species is less, the proportion of dominant flora is higher, and the diversity is lower. In the rank-abundance curve of the detailed samples (as shown in Figure 4B), we can see that the highest number and diversity of the flora are the sample GLP-2-116 of the test1 group, while the higher proportion of dominant flora and the lowest diversity are the sample DSS142 of the test2 group.

##### 3.4.2.2. Alpha diversity analysis

**Figure 5:**
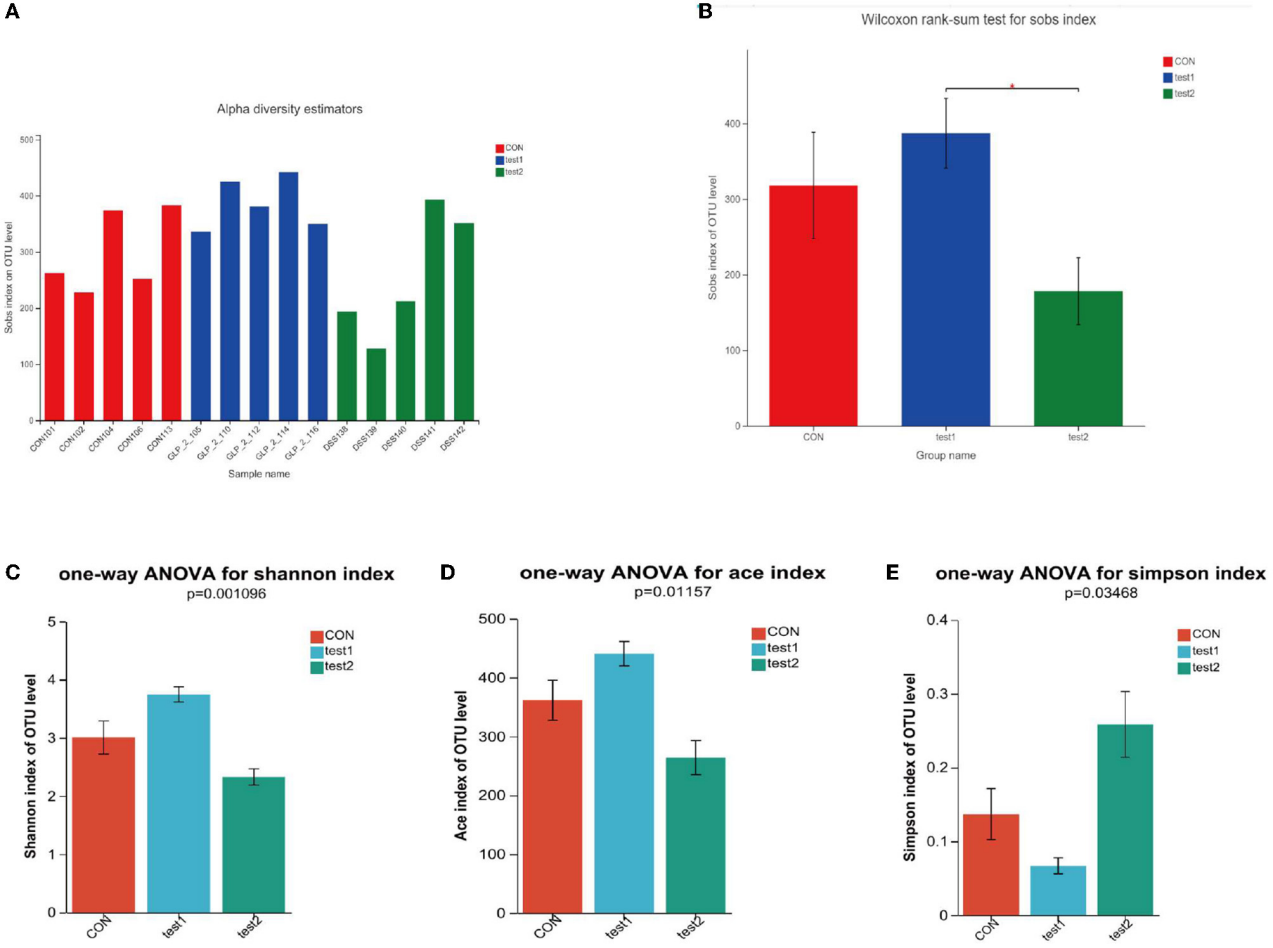
**(A)** Comparison of sample diversity among groups (Sobs index). **(B)** Diversity comparison Wilcoxon rank-sum test results. **(C)** Comparison of sample diversity among groups (Shannon index). **(D)** Comparison of sample diversity among groups (ACE index). **(E)** Comparison of sample diversity among groups (Simpson index). **(A)** Horizontal coordinate is the sample name, and the vertical coordinate is the number of OTU clusters for the colony at that grouping level. Type I intervals represent the upper and lower limits of the index. **(B)** *P < 0.05. **(C–E)** This figure shows the significant differences between the three selected groups of samples. The Abscissa is the group name, and the ordinate is the exponential average of each group.

The alpha diversity analysis allows us to obtain information on the composition of each sample species (in this study, we mainly refer to bacteria) and use different indices to respond to information on abundance (Sobs and Chao), coverage (coverage), and diversity (Shannon, ace, and Simpson). We performed comparisons between diversity samples for the Sobs indices and plotted diversity histograms as in Figure 5A and performed Wilcoxon rank-sum test for diversity between groups as in Figure 5B; at the same time, we also compare the diversity of samples among groups according to Shannon, Simpson, and ACE indices, such as Figures 5C–E. We found that the GLP-2 group had the highest sample diversity index and the DSS group had the lowest sample diversity index, and the difference was statistically significant.

**Figure 6:**
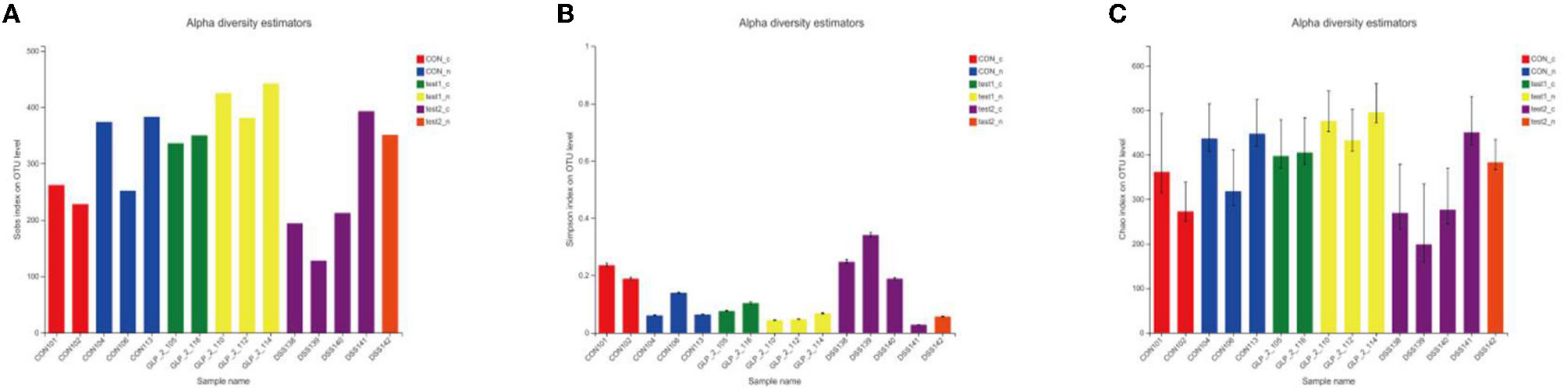
**(A)** Sobs index. **(B)** Simpson index. **(C)** Chao index.

Next, we compared the flora of the samples grouped by glycemic condition and found that in the three groups, the richness and diversity of the intestinal flora were reduced in the samples with elevated OGTT2h glucose compared to the samples with normal glucose, and in the intergroup comparison, as in the above results, the diversity of the flora was reduced in the DSS group compared to the control group, but the glycemic index was restored after the administration of GLP-2 intervention, the abundance and diversity were restored, and the between-group diversity tests of the flora were statistically different. Here, we used Sobs and Simpson index to assess the diversity, the higher the Sobs the lower the Simpson index the higher the diversity of the flora, and applied the Chao index to assess the richness of the flora. The results are shown in Figure 6.

#### 3.4.3. Analysis of the composition of the bacterial flora

##### 3.4.3.1. Venn diagram analysis

**Figure 7:**
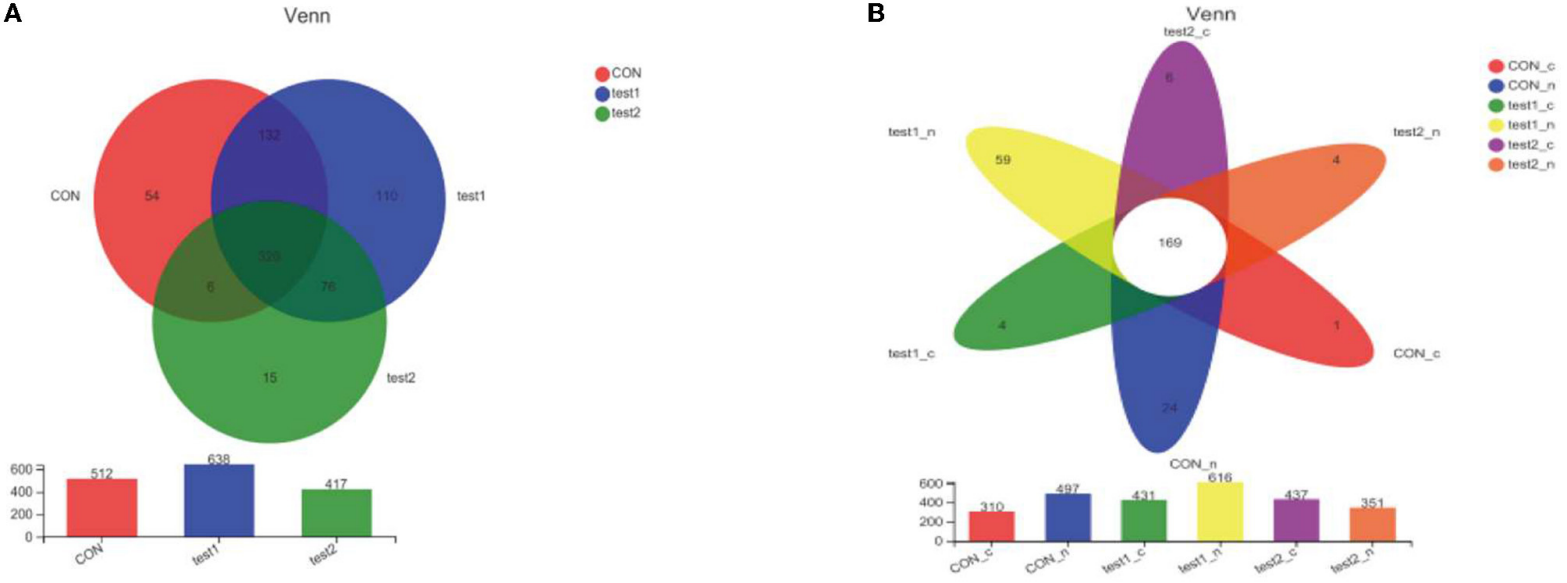
Venn diagram. The crossed parts of the colors represent the common species, and the non-crossed parts are the unique species of each group of samples. The bar chart below indicates the total number of bacteria. **(A)** Venn Diagram at the level of OTU in each group of mice. **(B)** Venn diagram at OTU level after grouping according to blood glucose in each group.

The number of common and unique species OTU in the control, DSS, and GLP-2 groups were counted, as shown in Figure 7A. We can visually see that the normal control group and GLP-2 group have more overlapping species, which means that the composition of the flora is closer. However, in terms of species composition, there are also more species unique to the GLP-2 group. An elevated diversity of flora was presented. In the grouping of glycemic conditions, we were able to see that the samples with abnormal blood glucose showed a decrease in bacterial species, but there was no appearance of specific flora, and the species were close to the samples with normal blood glucose in the same group, without significant differences in species composition, as shown in Figure 7B.

##### 3.4.3.2. Community bar graph analysis

**Figure 8:**
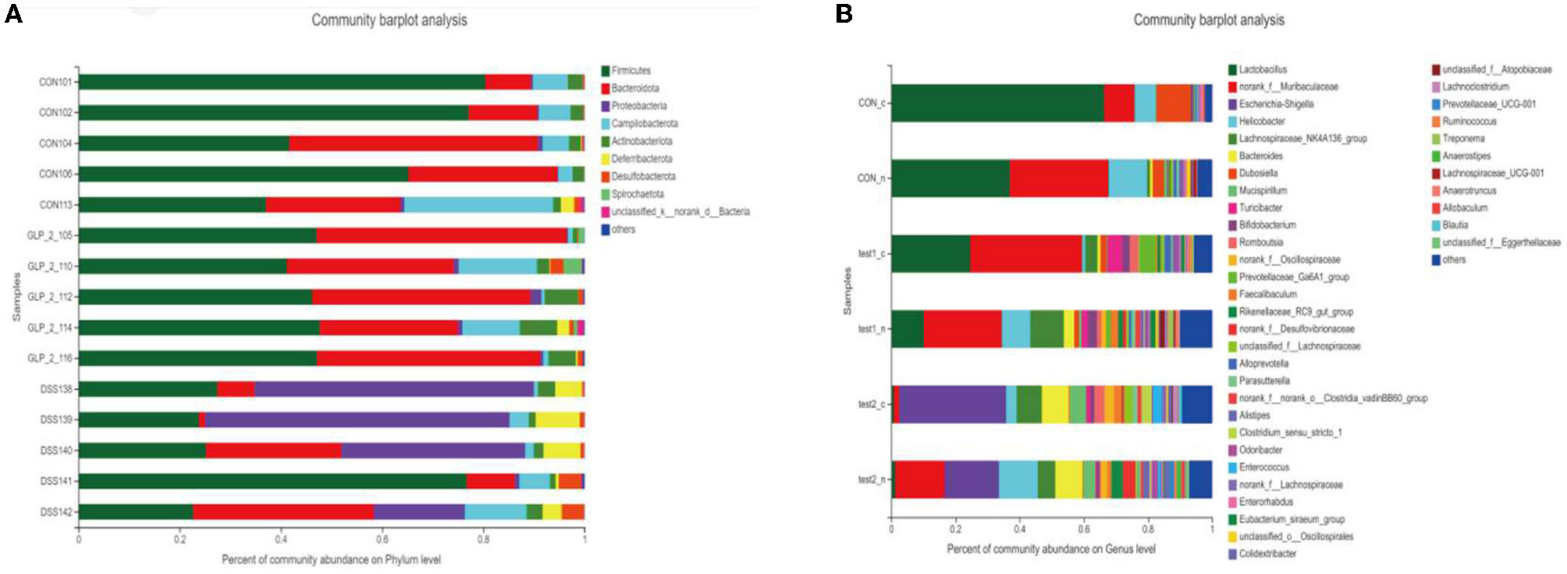
Column chart of the community. Vertical coordinate: sample name, horizontal coordinate: colony species and proportion (different color lengths are indicated). **(A)** Community barplot analysis in the detailed samples. **(B)** Community barplot analysis after grouping according to blood glucose in each group.

After performing taxonomic analysis, we obtained the community structure composition of the 15 samples at the taxonomic level of family, genus, species, OTU, etc. For a more visual representation, I also drew a Bar graph (as in Figure 8A), and we can see (1) the species of the flora of the samples; and (2) the relative abundance of the flora of the samples (the proportion occupied); we found that in the intestinal tract of mice bacterial composition, at the level of genera, Firmicutes was the dominant genus in the normal control group, while the proportion of Firmicutes decreased significantly after DSS modeling, showing the dominance of Proteobacteria genus, but after the administration of Glp-2 interference, Firmicutes recovered, but at the same time, the proportion of Bacteroidota also increased significantly. In the comparison of blood glucose grouping, the composition of the DSS group was significantly different from the control group both in terms of species and quantity; after the expected GLP-2 intervention, the species tended to recover toward the control group, but the quantity of the flora had not fully recovered, and the samples with relatively elevated blood glucose showed an increase in the abundance of Lactobacillus, but the overall flora was close to the same group of normoglycemic samples. The results are shown in Figure 8B.

##### 3.4.3.3. Community heatmap analysis

**Figure 9:**
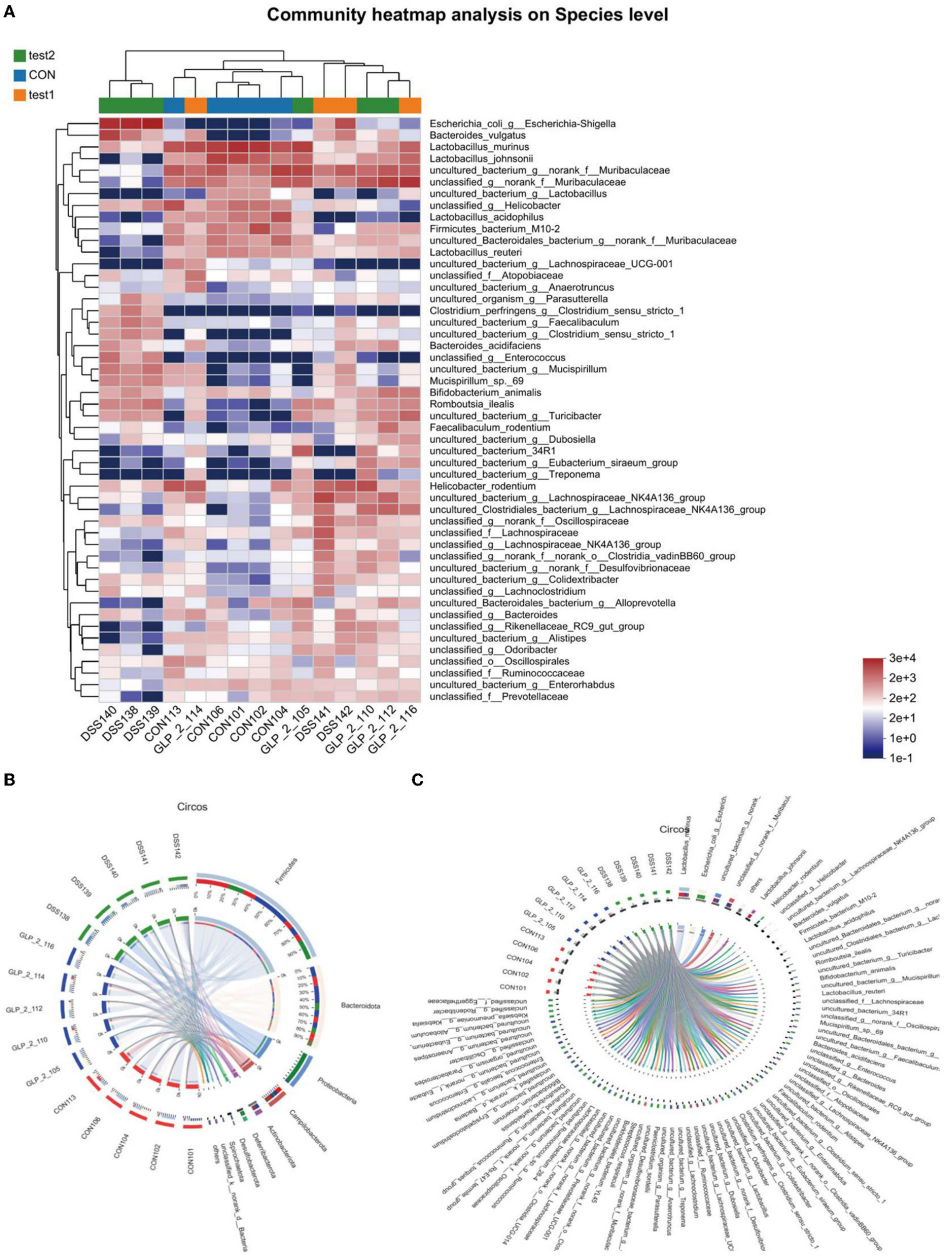
**(A)** Horizontal coordinates are the sample names, and the vertical coordinates are the bacterial names. Different colors represent different abundances, warm tones represent high abundance, and cool tones represent low abundance, and the abundance indices represented are marked in the lower right corner. **(B)** Distribution proportion of dominant species at the phylum level in each sample. **(C)** Distribution proportion of dominant species at the species level in each sample. **(B, C)** Circos sample–species relationship diagram shows the percentage of bacteria possessed by different samples and their distribution. Two circles, the inner circle is the sample name, and the outer circle is the colony name.

This map can especially visualize the information of the sample flora and the information of the difference of flora and can distinguish the abundance of species by color, which will show all the information of the sample flora and the difference and similarity between each sample in one place, as shown in the figure; at the level of species, the normal control group showed the high abundance of Lactobacillus_johnsonii and uncultured_bacterium_g__norank_f__Muribaculacea and unclassified g_ norank _f Muribaculaceae in high abundance, while Bacteroides_acidifa and unclassified _g Enterococcus uncultured_bacterium_g__Mucispirillum in low abundance. While the DSS group showed low abundance in the normal control high abundance group, Escherichia_coli_g__Escherichia-Shigella showed high abundance, and the GLP-2 group species abundance gradually approached the normal control group, but the abundance values were generally high. The results are shown in Figure 9A, while we reflected the proportion of dominant species distribution in each sample and the proportion of each dominant species distribution in different samples by visualizing circle plots at the phylum level in Figure 9B and at the species level in Figure 9C.

#### 3.4.4. Beta diversity analysis for sample comparison

##### 3.4.4.1. PCA analysis

**Figure 10:**
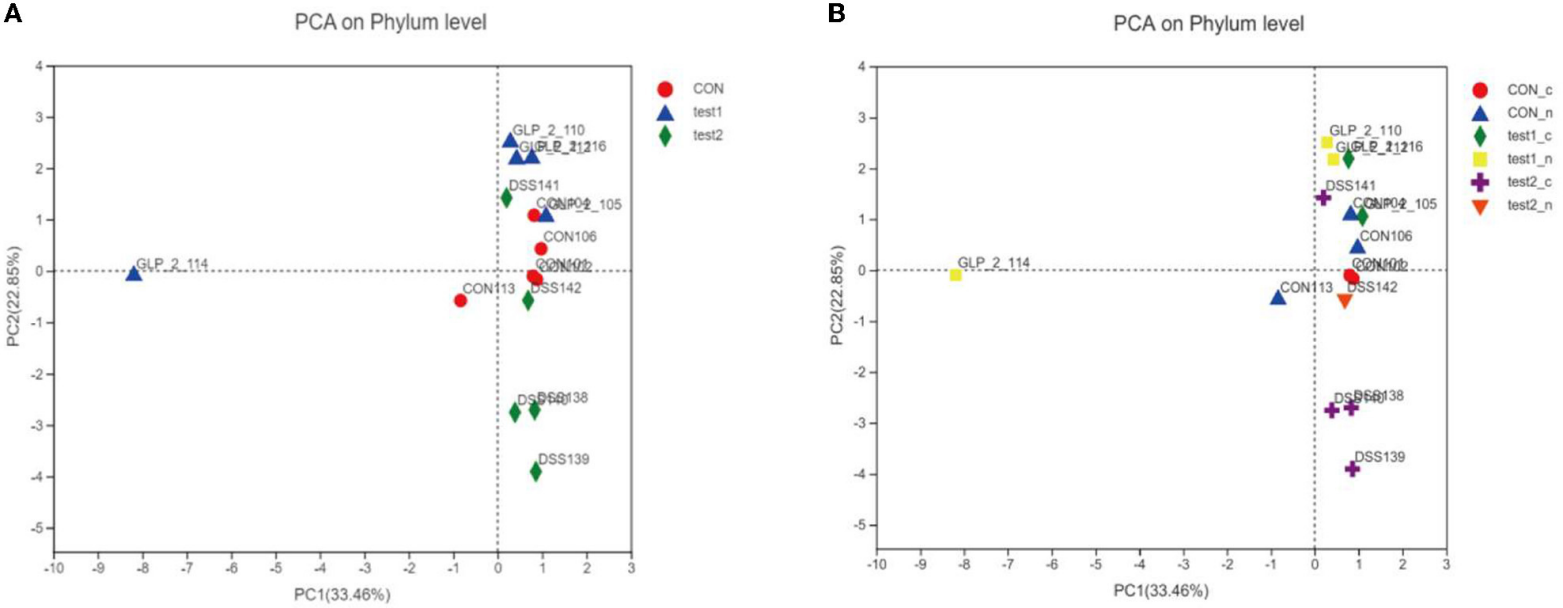
PCA analysis chart. Different colors and shapes represent different groupings. The more clustered the samples are on the axes, the smaller the differences in the populations, and conversely, the more discrete they are, the greater the differences in the number and composition of the populations. **(A)** PCA analysis chart in the group samples. **(B)** PCA analysis chart after grouping according to blood glucose in each group.

We plotted the flora of 15 samples in PCA, and we can intuitively conclude that, in the composition of the flora, the CON group and test2 group (GLP-2 group) are basically close, the coordinates are concentrated in the upper right limit, while the DSS group specimens flora composition coordinates are concentrated in the lower right limit, as shown in Figure 10A. According to the blood glucose grouping, we can intuitively see that the DSS group is relatively far away from the composition of the flora as shown in Figure 10B. GLP-2 interference and normal control group regardless of blood glucose and the composition of the flora are relatively close, and it can be seen that blood glucose has little effect on the composition of the flora, but the overall abundance of the flora affects the metabolic level of blood glucose.

#### 3.4.5. Variation analysis of species

**Figure 11:**
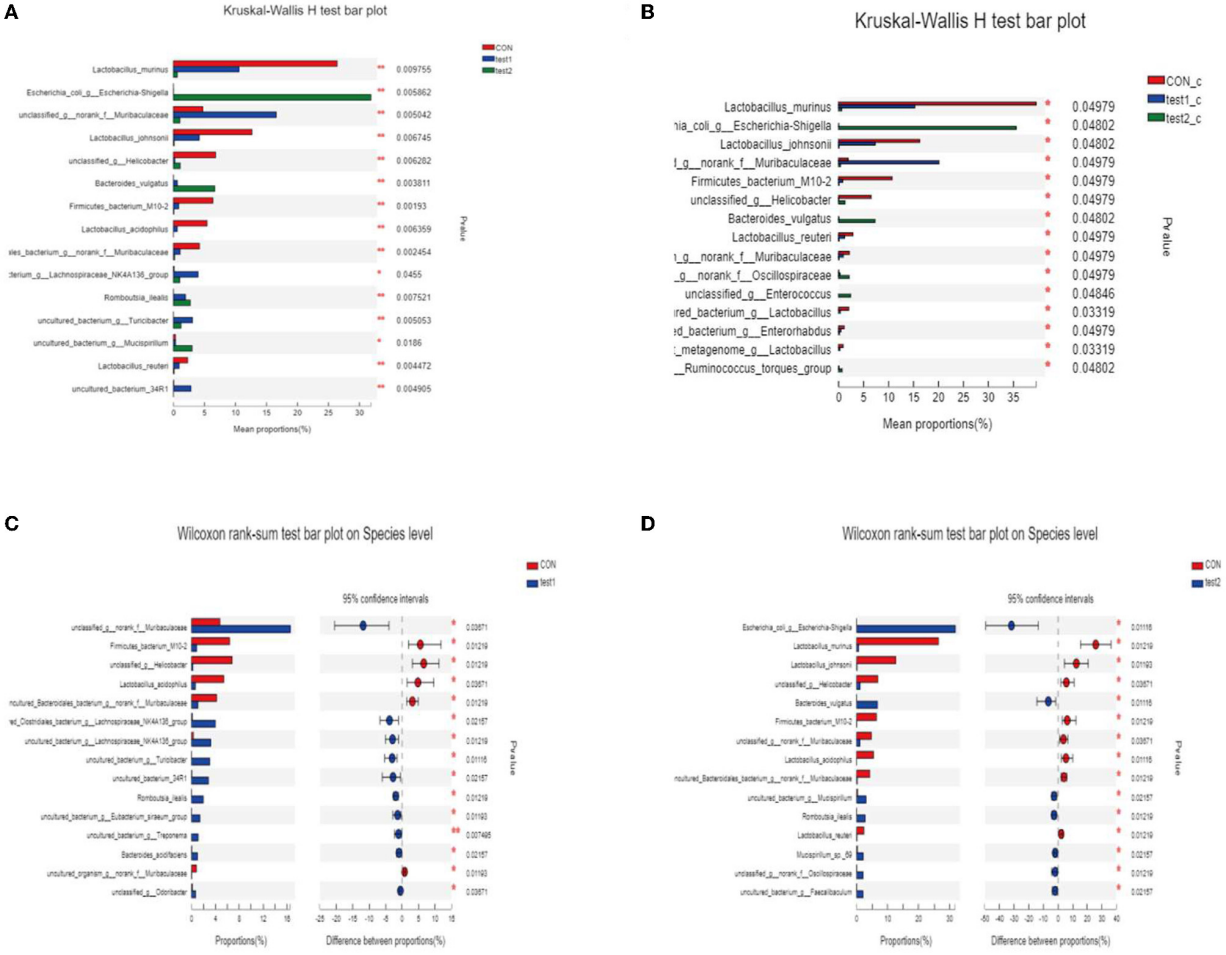
**(A, B)** Analysis of variance of species. *P < 0.05; **P < 0.01. **(C)** Statistical results of the difference in flora between the CON group and the test1 group. **(D)** Statistical results of the difference in flora between the CON group and the test2 group.

We subjected the obtained community abundance data to a significance test for differences between groups, and since we were comparing three groups, we used the Kruskal–Wallis rank-sum test. Through the following bar chart, we were able to find that Lactobacillus_murinus, Lactobacillus_johnsoniiun, classified_g__Helicobacter, and Lactobacillus_acidophilus were the dominant species in the normal control group, with statistical significance, and unclassified _g__ norank _f __ Muribaculaceae, etc. were the dominant strains in the GLP-2 group, while the abundance of the dominant strains in test2 (DSS group) was all lower. As shown in Figure 11A, the comparison between groups according to the grouping of abnormal blood glucose revealed no difference in species composition between normal and abnormal blood glucose in the samples under the same experimental conditions, which is consistent with the visual observation in the compositional analysis of the flora. However, in the samples with abnormal blood glucose, there were differences in the flora of the three groups with different experimental conditions, indicating that the degree of inflammation still directly affects the species of the flora and that abnormal metabolism of blood glucose exacerbates this difference. As shown in Figure 11B, the samples with abnormal blood glucose in the normal control group showed Lactobacillus murinus as the dominant species, Escherichia coli _g__ Escherichia-Shigella in the DSS group, while the GLP-2 group remained unclassified _g__ norank _f __ Muribaculaceae; the situation of their overall flora differences is basically the same as the overall grouping, which shows that the differences of flora basically appear in the samples with abnormal blood glucose. Next, we compared the experimental group with the control group between the two groups separately and applied the Kruskal–Wallis H rank-sum test, and the results obtained are shown in Figures 11C, D.

#### 3.4.6. Correlation analysis of flora and observation index

**Figure 12:**
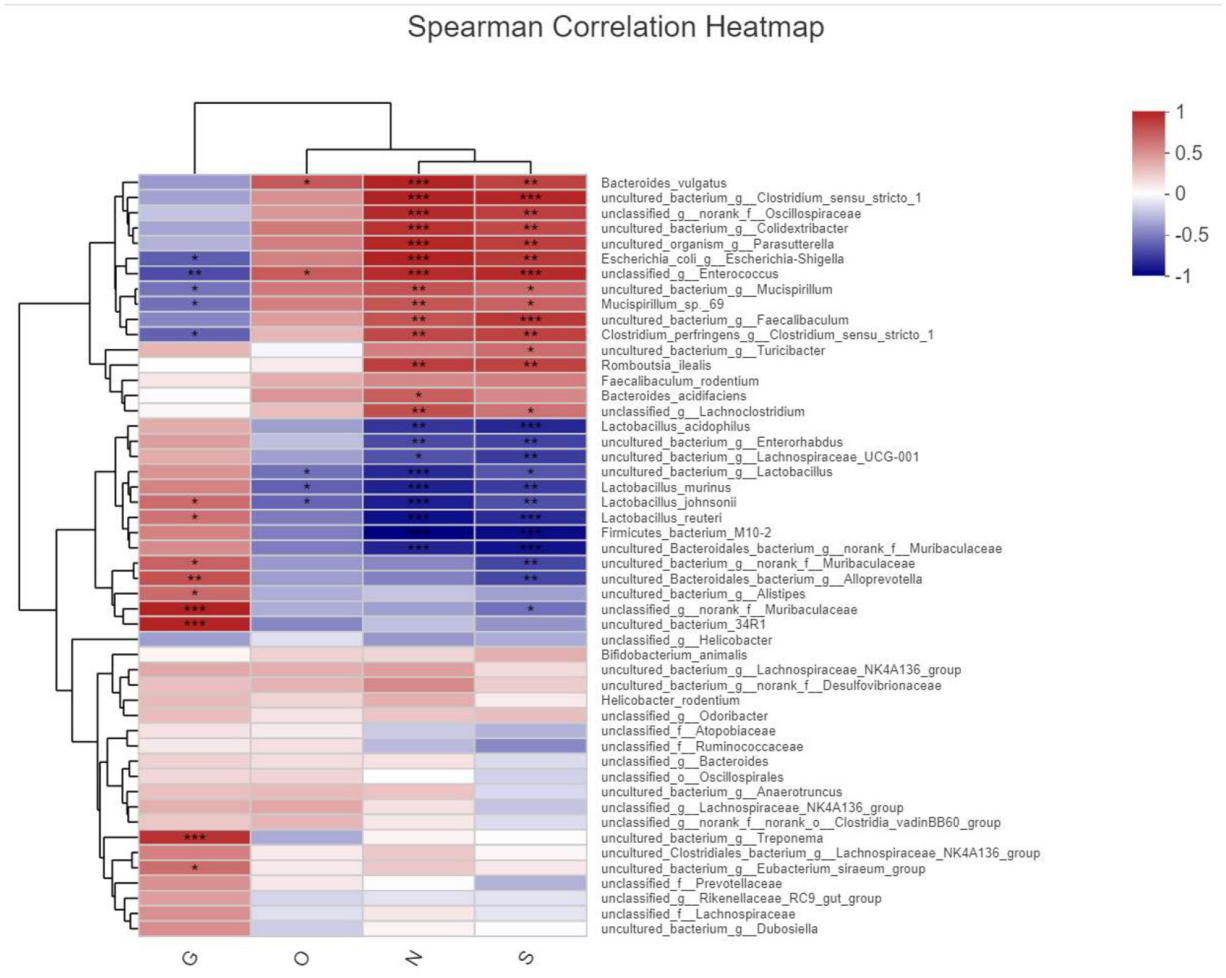
Heatmap plot of environmental factor correlations. X-axis is the observed index of this experimental study by name abbreviation labeled as (G, O, N, and S) and Y-axis bacterial name, different colors represent correlation, warm color represents large correlation, cold color correlation is small, and *represents the correlation of *P*-value, labeled the same as before.

This analysis is an important part of this experiment, and we analyzed the relationship between each environmental factor and the species and abundance of the flora in the samples by entering the environmental factors of the analysis system, the GLP-2 serum expression values (G), the 2-h glucose values (O), NF-κB (N), and STAT3 (S) serum expression values of the OGTT experiment, which were the main ones studied in this experiment. Correlations between the species abundance of intestinal flora and environmental variables were assessed. First, we observed the overall relationship between the sample flora and environmental factors by plotting the heatmap of environmental factor correlations, as shown in Figure 12.

We clearly found that the masses of bacteria associated with the expression of inflammatory pathway factors NF-κB(N) and STAT3(S) were more numerous and in higher abundance, such as Bacteroides_vulgatus, uncultured_bacterium, and unclassified_Oscillospiracea, and those associated with the expression of GLP-2 The dominant species associated with GLP-2 expression were uncultured_bacterium, Alistipesunclassified_Muribaculaceae, etc. The species directly associated with elevated blood glucose in the OGTT experiment were fewer and in low abundance, and the statistically significant ones here was unclassified_g__ Enterococcus.

**Figure 13:**
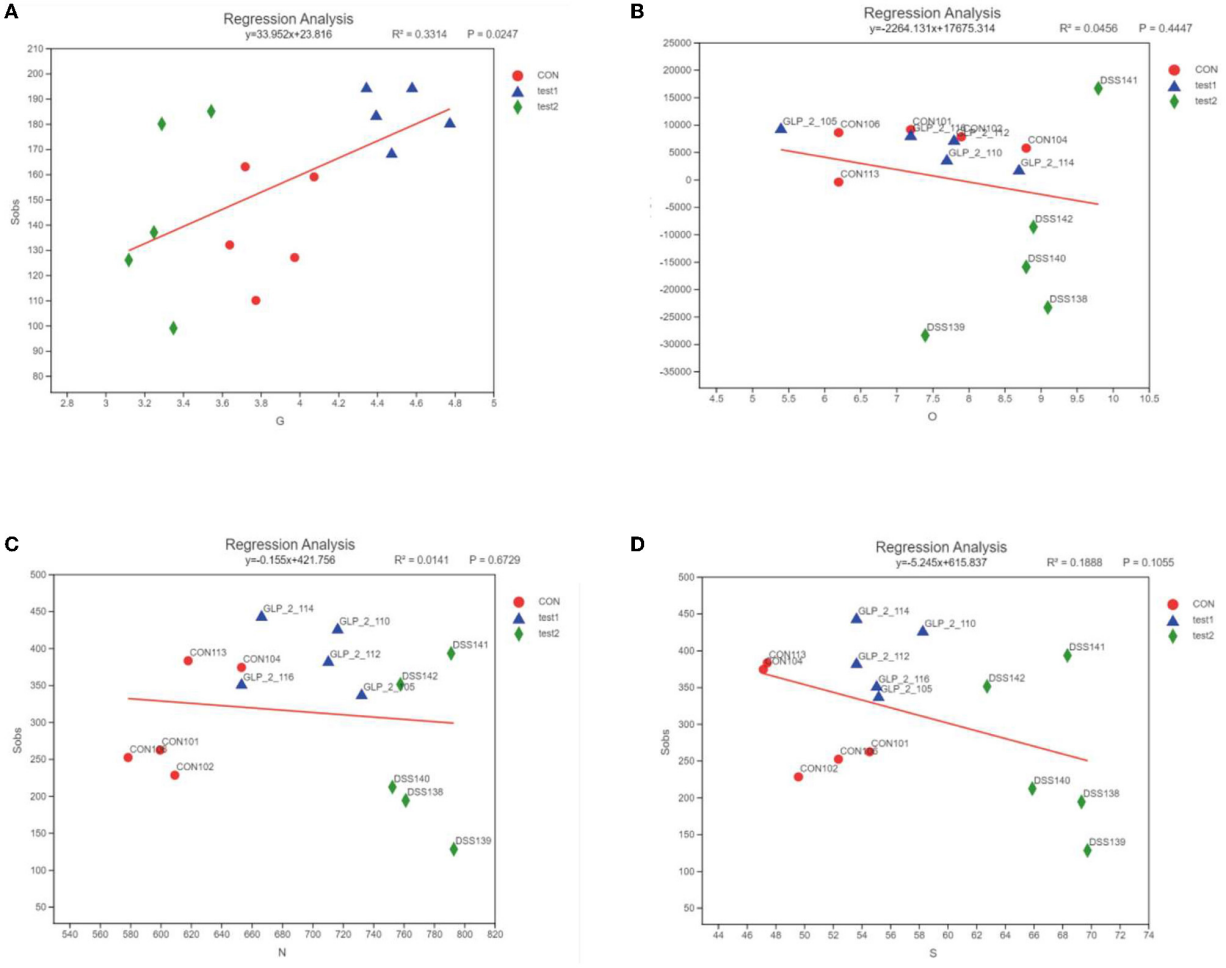
Linear regression results of the relationship between environmental factors and bacterial flora. The horizontal coordinates are the study indicators, and the vertical coordinates are the selected indices, here the Alpha diversity index; the slope of the red line represents the proportion of variation explained by the regression line, rising by the x-axis represents positive correlation, falling by the x-axis represents negative correlation. **(A)** GLP-2 and flora, **(B)** blood glucose and flora, **(C)** NF-κB and flora, and **(D)** STAT3 and flora.

In order to clarify the clear relationship between each indicator and the colony, we performed a linear analysis based on the results of PCoA analysis, making a scatter plot with the score of each sample on the PC1 axis as the y-axis and the environmental factors (G, O, N, and S) corresponding to that sample as the x-axis, and performing a linear regression, labeled R2, to evaluate the relationship between the two, where R2 is the coefficient of determination, which represents the proportion of variation explained by the regression line, as shown in Figure 13.

As shown in Figure 13, the expression of GLP-2 was positively correlated with the diversity of intestinal flora, the expression of blood glucose value at 2 h of OGTT test, NF-κB, and STAT3 was negatively correlated with the diversity of intestinal flora, the distribution of each experimental group was more concentrated, and the difference of DSS group was more obvious than the control group and GLP-2 group.

## 4. Discussion

UC is a chronic inflammatory disease characterized by recurrent and remitting inflammation of the mucosa of the colon and rectum. Its exact pathogenesis is not fully understood, but it is generally believed that a complex interplay of intestinal flora, genetic susceptibility, and environmental factors may disrupt the immune system, leading to an immune-mediated chronic intestinal inflammatory response (Deng et al., 2022). Prolonged inflammation leads to irreversible intestinal damage, which severely affects the quality of life of patients (Clooney et al., 2021). The main drugs currently used to treat UC include aminosalicylic acid, glucocorticoids, immunosuppressants, and biologics, and the potential side effects limit the use of current therapeutic agents (Zhou et al., 2022). Therefore, there is a need to find safer and more effective drugs for the treatment of UC. In the present study, we demonstrated that GLP-2 could reduce colonic inflammation by mechanisms including inhibition of NF-κB, JAK/STAT3 pathway exerting anti-inflammatory effects, regulation of glucose metabolism, and modulation of intestinal flora.

In this experiment, 2% DSS solution was used, and the mice in the modeling group gradually showed depression, laziness, weight loss, and positive occult blood from the 3rd day of modeling. Since clinical UC patients are often combined with infections of pathogenic bacteria, in this experiment, ETEC was added to the modeling to aggravate the intestinal inflammation of mice, to better simulate the changes of clinical UC patients and further investigate the therapeutic effect of GLP-2 on UC. ETEC, the most common causative agent of infectious diarrhea, produces a variety of non-heat and heat-resistant virulence factors, 26 adhesion factors that promote intestinal epithelial binding and colonization, and 3 enterotoxins responsible for humoral secretion, and these molecules may directly lead to the impairment of selective permeability tight junctions in intestinal tissues, thus disrupting the permeability barrier of intestinal epithelial cells (Dubreuil, 2017). It has also been shown that ETEC has a significant stimulatory effect on the expression of pro-inflammatory cytokines (IL-1α, IL-6, IL-18, and TNF-α), while there is no significant effect on the expression of anti-inflammatory cytokines (IL-4 and IL-10) (Bahrami et al., 2011). ETEC aggravated intestinal inflammation in mice in this experiment, as expected from the experiment.

In this experiment, the DSS and ETEC groups were also used as positive control groups, and in the disease activity index (DAI) score, colonic mucosal damage index CMDI score of the naked eye, and pathological histological score, the degree of inflammation was aggravated in the ETEC group compared with the DSS group, but in the ELISA results, the positive indexes in the ETEC group were lower than those in the DSS group, probably because ETEC aggravated the destruction of intestinal mucosa but did not dependent on NF-κB, JAK/STAT3 inflammatory pathway; it may also be due to the inadequate dose and number of ETEC administration, in animal experiments, in order to ensure that the survival rate of animals, ETEC dose, and interference time are conservative, which are the minimum standards of the reference literature, resulting in small differences in inflammation in some samples; on the basis of this experiment, our accumulated experience and deficiencies will be improved and supplemented in future experiments. Therefore, this experiment was mainly compared with the DSS group in exploring the mechanism of action of GLP-2.

After we interfered with the normal flora homeostasis of the mouse intestine with ETEC, both serological and histological GLP-2 expression decreased compared to the normal control while confirming the consequent increase in intestinal inflammation, suggesting that intestinal flora can modulate or indirectly affect GLP-2 expression, but at this point there was no statistical difference in GLP-2 expression levels compared to the ETEC group even after the administration of exogenous GLP-2. We analyzed that in the case of very heavy intestinal inflammation, the basal secretion of GLP-2 is very little due to the absence of intestinal epithelium, so it cannot show the effect in the case of short time interference (Salaga et al., 2017), and we will improve it in the next experiments to increase the treatment time of exogenous GLP-2 and verify the experimental results.

GLP-2, as an enterotrophic hormone secreted by intestinal endocrine L cells, is rapidly degraded by DPP-IV *in vivo* with an extremely short half-life. In the clinic, GLP-2 analogs are mainly administered by subcutaneous injection, and patient compliance is poor, so this experiment chose the DPP-IV inhibitor sitagliptin to indirectly increase the level of GLP-2 by gavage in mice, and for the administration of sitagliptin, the following points are explained: Intraperitoneal injection is the injection of the drug into the animal's gastrointestinal tract outside the plasma membrane and within the peritoneum and is generally used for administration where absorption through the gastrointestinal tract is inappropriate or less efficient. Gavage administration refers to the direct instillation of drug solution into the stomach of animals with the help of a gavage needle, which can achieve precise drug administration and flexible control of drug dose, and can also well simulate some *in vivo* effects of drugs after oral administration in clinical settings. In this experiment, sitagliptin was administered by gavage instead of intraperitoneal injection for the following two reasons: On the one hand, since the route of administration of sitagliptin in clinical practice is oral, gavage in mice can better analyze the drug efficacy in clinical patients; on the other hand, GLP-2 is released from intestinal endocrine L cells and later degraded by DPP enzymes, and sitagliptin can better play the role of inhibiting the degradation of GLP-2 by gastrointestinal administration. However, sitagliptin can also prolong the half-life of GLP-1, and it is worthwhile to investigate whether GLP-1 plays a role in the intestinal tract in addition to its known role in regulating blood glucose.

### 4.1. GLP-2 reduces the degree of inflammation in UC mice

GLP-2 is an intestine-derived hormone that promotes intestinal growth, digestion, absorption, barrier function, and blood flow in healthy animals, prevents damage, and promotes repair in preclinical models of colitis, and after massive small bowel resection (Li and Weigmann, 2022), GLP-2 expression is reduced in UC due to the destruction or inhibition of intestinal endocrine L cells in the inflammatory state (Zatorski et al., 2019). Exogenous administration of GLP-2 has been shown to be effective in reducing the symptoms of intestinal injury in animal models (Wu et al., 2015). In this experiment, mice in the GLP-2 group showed reduced blood stool condition, degree of weight loss, degree of carotid ulceration, and pathological histological score compared with mice in the DSS and ETEC groups, demonstrating the therapeutic effect of GLP-2 on UC. Ning et al. (2020) demonstrated experimentally that sitagliptin attenuates DSS-induced experimental colitis, and its effects can be attributed to increased GLP-2 expression and subsequent protection of the intestinal barrier by inhibiting epithelial cell apoptosis and promoting its proliferation. Sitagliptin, a DPP-IV inhibitor, prolongs the half-life of GLP-2, attenuates intestinal inflammation in UC mice, and is expected to be a new drug for the treatment of UC.

### 4.2. GLP-2 inhibits NF-κB pathway

Among immunomodulatory factors, the inflammatory response is considered a central mechanism in the pathophysiology of UC, and pro-inflammatory cytokines play an active role in the inflammatory response by inducing macrophage migration and the release of inflammatory mediators, which further amplify the inflammatory response (Zhao et al., 2022). NF-κB plays a central role in regulating the inflammatory process (Lee et al., 2022). Activation of NF-κB has been reported to upregulate the expression of pro-inflammatory cytokines that trigger positive feedback regulation during inflammatory activation, ultimately damaging colonic tissue (Pandurangan et al., 2022). To further investigate the anti-inflammatory mechanism of GLP-2, the expression of NF-κB p65, an NF-κB-related protein, was examined in colonic tissues in this experiment. The results showed that NF-κB expression was significantly higher in the DSS group compared with the control group, due to the fact that DSS aggravated intestinal mucosal damage and increased intestinal inflammation in mice, activating the NF-κB inflammatory pathway, consistent with previous studies (Gu et al., 2022). Compared with the DSS group, NF-κB expression in both serum and colonic tissues of mice in the GLP-2 group was significantly reduced, and Xie et al. (2014) demonstrated that GLP-2 significantly reduced lipopolysaccharide-induced production of inducible nitric oxide synthase (iNOS), cyclooxygenase-2 (COX-2), IL-1β, IL-6, and tumor necrosis factor-α (TNF-α). Signaling pathway analysis showed that GLP-2 reduced LPS-induced phosphorylation of NF-κBp65, consistent with the present experimental study, suggesting that GLP-2 may reduce inflammation by attenuating NF-κB activation. NF-κB controls the production and secretion of multiple cytokines and chemokines during UC pathophysiology, and it is a central inflammatory mediator induced by pro-inflammatory genes in both natural and acquired immune cells, which can respond to multiple immune receptors (Yao et al., 2019). Uncontrolled NF-κB activation is a hallmark of chronic inflammatory diseases, and targeting the NF-κB signaling pathway is an attractive anti-inflammatory therapeutic approach (Liu et al., 2017).

### 4.3. GLP-2 inhibits the JAK/STAT3 pathway

Cytokines are key mediators of inflammation-mediated imbalance in UC intestinal homeostasis and pathological processes. Most cytokines in UC such as IL-6, IL-10, IL-2, or IL-22, as well as those thought to be mediators of UC pathological responses (IFN-γ, IL-12, IL-23, or IL-9) act through the JAK/STAT3 pathway (Moon et al., 2021). Activation of STAT3 activates NF-κB, which enters the nucleus, binds to the promoters of target genes, and induces pro-inflammatory mediators such as iNOS, COX-2, TNF-α, and IL-6, which reactivates the JAK/STAT3 pathway, resulting in a crosstalk of STAT3-NF-κB-IL-6-STAT3, creating a malignant pro-inflammatory cycle, and allowing the disease to progress toward inflammatory cancer transformation (Fan et al., 2013). Blocking the JAK/STAT pathway has the potential to affect the complex inflammation driven by multiple cytokines associated with UC pathology compared to more traditional approaches using antibodies to block single cytokines (Salas et al., 2020). In this experiment, both serum and colonic tissue STAT3 were significantly higher in the DSS group of mice than in the blank group, and both serum and colonic STAT3 were significantly lower in the GLP-2 group compared to the DSS group, with serum IL-6 showing the same trend. Ivory et al. (2008) investigated the mechanism of anti-inflammatory effect of GLP-2 through an IL-10-deficient colitis mouse model and showed that the anti-inflammatory effect of GLP-2 was not dependent on IL-10 but was attributed to GLP-2 antagonizing IL-6-mediated STAT3 signaling, thereby inhibiting intestinal inflammation, consistent with the results of this experiment. GLP-2 may also reduce the pro-inflammatory by inhibiting the expression of NF-κB mediator IL-6 release, blocking the JAK/STAT3 pathway and thus exerting an inhibitory effect on inflammation.

### 4.4. GLP-2 regulates the diversity and species composition of intestinal flora

Disturbances in the interaction between the intestinal flora and the mucosal immune system play a key role in the development of UC and are usually associated with reduced flora diversity and imbalances in strain composition (Bunt et al., 2021). Dysbiosis usually leads to a decrease in the production of short-chain fatty acids, which reduces an important source of energy for intestinal epithelial cells, leading to increased intestinal permeability and triggering inflammation (Wu et al., 2020). Compared to healthy intestinal flora, UC intestinal flora generally has lower taxonomic diversity and lower phylum levels (Shen et al., 2018). It is unclear whether the reported changes in intestinal flora are a cause or a consequence of UC. In this experiment, 16SrRNA assay was performed on mouse colonic tissues, and the results showed that the intestinal flora diversity was reduced in the DSS group, and the dominant bacteria such as Lactobacillus and norank_f__Muribaculaceae were significantly reduced; the inferior bacteria such as Escherichia-Shigella, Mucispirillum Clostridium_sensu_stricto_1, Romboutsia, Enterococcus, and Faecalibaculum significantly increased. Compared with the DSS group, the diversity of intestinal flora was increased, and the dominant bacterial norank_f__Muribaculaceae, Lactobacillus, and Prevotellaceae_Ga6A1_group increased significantly. Inferior bacteria Escherichia-Shigella, Mucispirillum, Enterococcus, etc. were significantly reduced. Jang et al. (2019) showed that Lactobacillus fermentum could improve dextran sulfate sodium-induced colitis by modulating immune response and altering intestinal flora. Cani et al. (2009) showed that prebiotics improve intestinal flora and mucosal barrier function by increasing endogenous GLP-2 production and that GLP-2 improves intestinals permeability and reduces plasma LPS levels by regulating the expression of the tight junction proteins zonula occludens-1 (ZO-1) and occludin, thereby blunting inflammation and oxidative stress. Li et al. (2021) demonstrated that GLP-2 improves colonizing bacteria and reduces the severity of UC by enhancing the diversity and abundance of intestinal mucosa, all of which corroborate the results of this study. The association analysis of GLP-2, NF-κB, STAT3, and bacterial flora in this experiment showed that the intestinal flora diversity increased with the increase of GLP-2 expression, so GLP-2 may play a protective role in the intestine by regulating the flora diversity and increasing the dominant species such as Lactobacillus.

In addition, we dynamically observed the changes of intestinal flora by aggravating and reducing intestinal inflammation, and it can be determined that the abundance and species of intestinal flora were negatively correlated with inflammation, but the abundance of intestinal flora recovered after inflammation, the diversity of flora increased depending on the treatment interference factors, and the species of dominant bacteria also differed slightly, for example, we gave GLP-2 interference after modeling, and normal. In the control group, Firmicutes (phylum thick-walled) was the dominant genus, while the proportion of Firmicutes decreased significantly after DSS modeling, showing the dominance of Proteobacteria genus, but Firmicutes recovered after Glp-2 interference, but at the same time, the proportion of Bacteroidota (phylum Bacteroidota) also increased significantly. In the beta diversity analysis, we compared the species of Bacteroidota in each sample and found that although there were differences in the dominant species after GLP-2 (positive interference) interference in DSS mice, the overall abundance and diversity of species were very similar to those of the normal control group, which means that we can assume that the species and abundance of intestinal flora will also tend to normalize when the intestinal inflammation gradually recovers, which is less affected by the therapeutic drugs and is a rather special conclusion of this experiment. In the analysis of the variability of the flora of the inflamed intestine, because the abundance of the flora of the modeling group (DSS group) was all very low, in comparison with the control and the control, Lactobacillus_murinus, Lactobacillus_johnsoniiun, and classified_g__Helicobacter Lactobacillus_acidophilus were all significantly different in terms of reduction, while the GLP-2 group showed statistical differences with the normal control Bacteroides_vulgatus and other species, no literature support for the relevant results was found, and we will further test and verify in subsequent experiments.

### 4.5. GLP-2 regulates glucose metabolism

For DSS-molded mice, we artificially aggravated the intestinal inflammation, and the results showed that the abnormal glucose metabolism of the mice was also statistically significant compared with the control group. Meanwhile, in the GLP-2 treatment group, we confirmed that the blood glucose level of the mice was restored after restoring the diversity and abundance of some intestinal flora by sequencing. It was demonstrated that changes in intestinal flora diversity and abundance had consistent effects on intestinal inflammation and glucose abnormalities and could be regulated simultaneously. On the contrary, after we gave the treatment with exogenous GLP-2, the expression of GLP-2 in mice increased and the blood glucose abnormalities were corrected, the diversity and abundance of the same flora were restored and showed a dominant flora such as Lactobacillus, as described previously, which was very close to the normal control group, and the results of the changes of flora and blood glucose in the experiment were also consistent with the results of many diabetic modeling mice with intestinal. The results of the experimental flora and blood glucose changes are also in comparison with the results of inflammatory intestinal flora in many diabetic modeling mice (Wang F. et al., 2023). This result fully illustrates that the regulation of glucose metabolism and flora is bi-directional and correlates with the degree of inflammation in the intestine. It is believed that with the further analysis and precision of the flora species, we can find the superior monobacteria that can control the intestinal inflammation and adjust the blood glucose level at the same time.

In the analysis of intestinal flora, the abundance and diversity of the flora of the modeled mice treated with exogenous GLP-2 were very close to those of the normal control group, confirming that the increase of the protective factor GLP-2 could reduce intestinal inflammation and restore the intestinal environment, which is consistent with the results of studies related to the relationship between intestinal flora and GLP-2 factor (Cani et al., 2009). In terms of glucose metabolism regulation, the GLP-2 group showed an absolute advantage, which means that the role of GLP-2 in the glucose metabolism of mice is clear, we gave exogenous GLP-2 treatment, serological ELISA confirmed the upregulation of GLP-2 expression, drug interference was effective, and the 2-h blood glucose level in the OGTT experiment was significantly different in the GLP-2 group compared to the DSS group and the toxin-producing E. coli group, while there was no statistical difference with the normal control group, indicating the effective recovery of blood glucose levels, which is consistent with the experimental findings of Cani et al. (2007) that the blood glucose levels in obese mice with combined type 2 diabetes were effectively reduced by increasing the GLP-2 content in mice. It is worth affirming that GLP-2 is beneficial for intestinal inflammation, flora environment, and metabolism of blood glucose, and since GLP-2 is a short peptide chain containing only 33 amino acids and is very easy to obtain chemically, in future, exogenous supplementation of the protective secretory factor GLP-2 could then provide new therapeutic ideas for UC and its associated metabolic diseases.

## 5. Conclusion

In summary, GLP-2 was able to significantly reduce the clinical symptoms in UC mice and significantly reduce the DAI score, visual CMDI score, HE staining, and pathological histological score, which reflect the degree of inflammation, even though the intestinal inflammation was aggravated by ETEC infection in UC mice. This experiment tentatively demonstrated that GLP-2 may block the malignant pro-inflammatory cycle by inhibiting NF-κB pathway and JAK/STAT3 inflammatory pathway, regulating glucose metabolism, and exerting intestinal protective effects by increasing the dominant strain and regulating flora diversity.

## Data availability statement

The datasets presented in this study can be found in online repositories. The names of the repository/repositories and accession number(s) can be found in the article/supplementary material.

## Ethics statement

The animal study was reviewed and approved by the Animal Ethics Committee of the First Hospital of Harbin Medical University.

## Author contributions

DL and HX designed the study. YG wrote the manuscript. DL revised the manuscript. YG, LC, YL, HL, and XT carried out the experiments. All authors read and approved the final manuscript.
